# Effect of Addition of Different Phenolic-Rich Extracts on Beer Flavour Stability

**DOI:** 10.3390/foods9111638

**Published:** 2020-11-10

**Authors:** Giovanni De Francesco, Elisabetta Bravi, Emmanuel Sanarica, Ombretta Marconi, Federica Cappelletti, Giuseppe Perretti

**Affiliations:** 1 Italian Brewing Research Centre, University of Perugia, 06126 Perugia, Italy; gio.defrancesco@hotmail.it (G.D.F.); e_bravi@yahoo.it (E.B.); giuseppe.perretti@unipg.it (G.P.); 2 Enolife s.r.l., Viale delle Industrie, Montemesola, 74020 Taranto, Italy; emmanuelsanarica@enolife.it; 3 Department of Agricultural, Food and Environmental Science, University of Perugia, 06126 Perugia, Italy; cappellettifederica@ymail.com

**Keywords:** beer quality, phenolic extract, beer flavour stability, condensed tannins, antioxidant, ABTS, FRAP, DPPH, tea tannins, grape seed tannins, beer shelf life

## Abstract

Flavour stability is a key factor in the beer production process. The stabilizing effect of six commercial phenolic-rich extracts was studied. The extracts were added to beer before bottling. Quality parameters (colour, turbidity, foam and dissolved oxygen content), antioxidant activity by 2,2′-Azino-bis(3-ethylbenzothiazoline-6-sulfonic acid) (ABTS), ferric reducing antioxidant power (FRAP), 1,1-diphenyl-2-picrylhydrazyl radical (DPPH), total polyphenols content, and sensorial analysis by a trained panel were performed over the course of storage. The beers were analyzed every 3 months for a total period of 6 months. Results indicated that all studied phenolic-rich extracts positively affected the beer flavour stability. In particular, the condensed tannins showed a significant protective effect. The condensed green tea tannins resulted as the most promising source of natural antioxidant able to prolong beer shelf-life and bring interesting organoleptic characteristics to beer. Also, grape seed tannins appeared suitable to boost flavour stability and improve organoleptic properties of beer.

## 1. Introduction

The preservation of beer quality throughout its lifetime is still a considerable challenge for brewers. Beer quality is typically determined by microbiological, colloidal, foam, colour, and flavour stabilities during time. Nowadays, the trouble of appearance of hazes and the growth of micro-organisms in beer is largely under good control [1,2].

Currently, the flavour stability is the most studied topic and perhaps the most important quality parameter of beer industry. Moreover, the consumer could even appreciate the flavour of an aged beer; anyway, the expectation of consumers to recognize the flavour of that particular brand of beer they usually drink and the constancy of that flavour is far more important [2,3]. However, considering that the flavour recognized by the consumer is normally the flavour of the fresh beer, staling become undesirable and, as much as possible, must be avoided or at least prolonged [2].

Beer aging is a very complex phenomenon connected to changes in chemical constituents. Since several years ago, it was assumed that trans-2-nonenal was mainly recognized as a marker compound of beer staling. Today, many compounds have been discovered to be involved in off-flavour or flavour variation during staling of beer [4].

Beer flavour stability is influenced by several factors, some of them are disputed, but the oxidation certainly plays a crucial role in flavour [2]. Minimizing the formation and activity of reactive oxygen species (ROS: O_2_, HOO•, H_2_O_2_ and HO•) in beer and wort is definitely a first step to get better beer flavour stability [2,5]. 

It has been well demonstrated that high O_2_ levels into final package reduce the shelf-life of beer [6]. Moreover, the presence of transition metal ions (Cu^+^ and Fe^2+^) promotes the formation of ROS acting as electron donors. Consequently, process and technological parameters should be improved to minimize wort and beer oxygen pick-up as well as low amount of copper and iron. Modern fillers are designed to keep oxygen level in the packaged beer as low as possible, aiming for a maximum pick-up of 100 ppb during filling. Moreover, antioxidants may be used in the beer (depending on the local legislation), especially sulphur dioxide and ascorbic acid [5,7,8]. 

Beer flavour changes during storage as a result of an increase in the amount of several compounds among which the most significant are carbonyl compounds mainly generated via oxidation of higher alcohols, autoxidation of unsaturated fatty acids, enzymatic degradation of unsaturated fatty acids, Maillard reactions, and oxidative degradation of isohumulones. This process can be greatly slowed down by the addition of substances that act against the oxidation (antioxidants) [7].

In wort and beer there are many endogenous molecules with antioxidant or prooxidant activities, such as sulphites, polyphenols, melanoidins, chelating agents, and lipoxygenases [2]. Phenolic compounds show important antioxidant activity in many natural systems. Lower molecular weight polyphenols are excellent antioxidants [9,10,11]. 

In beer, the polyphenol fraction originates from barley malt (70–80%) and from hop (20–30%) [12,13]. 

Polyphenols antioxidant activity is related to their capacity to react with free radicals to produce phenoxy radicals and to their ability to chelate transition metal ions [9,14,15,16].

The present study aimed to evaluate the protective effect of the addition of different phenolic extracts on beer stability and quality. Six commercial phenolic extracts were added to the beer at the bottling. The added extracts were: (i) hydrolysable phenolic extracts, commercial names “beer tannin-gallotannin” (BTGT) and Oxnomore mash; (ii) condensed phenolic extracts, commercial names Oxnomore pro, Oxnomore super and “beer quebracho tannin” BTQ; (iii) combination between hydrolysable and condensed, commercial name “beer condensed tannin” (BTC) (Table 1).

Qualitative parameters related to the oxidation and potential interaction between polyphenols and protein were chosen. Apparent attenuation, colour, turbidity, foam, dissolved oxygen content, total polyphenols content, antioxidant activity (by the three assays ABTS, FRAP, DPPH), and sensorial analysis by a trained panel were performed every 3 months, for a total time of 6 months, starting from the end of refermentation. The ABTS, FRAP and DPPH tests indicate that hydrolysable tannins should have a higher antioxidant capacity than condensed tannins in wine model solution. To our best knowledge, there are no works on beer that compared hydrolysable to condensed tannins in beer [17].

## 2. Materials and Methods

### 2.1. Wort and Beer Production

The production of the wort and beer was carried out at the 100 L pilot plant of the Italian Brewing Research Centre of the University of Perugia (Italy).

A target of 12.5 °Plato was obtained from 100% Pilsner malt (Bestmalz, Heidelberg, Germany) ground with a double roller mill, the distance of which was adjusted to 1.1 mm.

The mashing and sparging water were obtained by mixing 20% of tap water and 80% of osmosis water and the water/malt ratio of 3:1. Ten grams of calcium chloride and calcium sulphate in mash and 15 g in the boiling phase were added and 19 mL of lactic acid 80% to decrease a pH of boiled wort of 5.20. The mashing temperature was set at 65 °C for 45 minutes and subsequently brought to 72 °C for 20 minutes. After saccharification, the temperature was raised to 78 °C to mash out and then filtrate. For the boiling phase, Cascade hops were added 60 and 10 min before the end of the boil to obtain the target of 20 IBU (International Bitterness Unit). Five grams of CLARBREW C (Enolife srl, Montemesola, Italy) to improve the removal of tannin-protein complexes was added at the end of boiling. The wort obtained was cooled and pitched with Safale US-05 (Fermentis, Marcq-en-Baroeul, France). The fermentation temperature was fixed at 21 °C until the attenuation limit was reached. At the end of fermentation, the temperature was lowered to allow the yeast to settle down and clarify the beer. 

Finally, each beer sample was bottle conditioned at 20 °C for 2 weeks with a sucrose:water solution (w:w 1:1) to yield 5.5 g/L of CO_2_ in the bottle. During the bottling step, 20 g/hL of each phenolic-rich extract (listed in Table 1) were added to the beer, according to the guidelines of the producer. All the samples were analysed immediately starting from the end of refermentation (fresh beer, M0) and after 3 and 6 months (M3 and M6) of storage at room temperature (22–25 °C). All experiments were performed in duplicate. 

### 2.2. Standard Quality Attributes

Beer analysis was performed every 3 months. The following standard Analytica European Brewery Convention (EBC) procedures (EBC, 2007) were performed: colour of beer by spectrophotometric method (9.6); foam stability of beer using the NIBEM-T Meter 30 s (9.42); haze in beer by haze meters (9.29). Apparent degree of fermentation was measured according to MEBAK method 2.8.4 (MEBAK, 2013). Dissolved oxygen in bottled beer is determined by an internal method using the Haffmans Inpack TPO/CO_2_ meter Type c-TPO (Pentair Food & Beverage Marssteden 50, 7547 TC Enschede, The Netherlands).

### 2.3. Phenolic-Rich Extracts

The phenolic-rich extracts under study were provided by the company Enolife srl (Montemesola, Taranto, Italy) and are represented by six commercial products based on phenolic extracts of different origins (Table 1).

### 2.4. Determination of Total Polyphenols Content

The Folin–Ciocalteu method [18] was applied to determine the total polyphenol content of free and bound fractions. Two milliliters of Folin–Ciocalteau reagent were added to 0.4 mL of extracts, then 1.6 mL of a 7.5% Na_2_CO_3_ solution was added. The obtained solution was incubated, at room temperature, in the dark for two 120 minutes. The absorbance of the mixture was measured at 760 nm. Standard solutions of Gallic acid (GA) were used to calibrate the method, and the content of total polyphenols was expressed as mg of GA equivalent (GAE) per g of sample dry weight (mg GAE/g DW).

### 2.5. Antioxidant Activity Measurement

Antioxidant activity describes the ability of redox molecules, in foods or biological systems, to scavenge free radicals and to reduce oxidant compounds. In this study, the antioxidant activity of beers was measured by ABTS, DPPH and FRAP tests, using Trolox as a standard for the calibration curves [19]. For ABTS, an aliquot (150 μL) of beer was mixed with the ABTS• + solution (2850 μL), and the absorbance was read at 734 nm after 2 h in the dark. Before use, the ABTS• + solution was prepared by mixing equal amounts of stock solutions of 7.4 mM ABTS• + and 2.6 mM potassium persulfate for 12 h and the solution was then diluted adding methanol to obtain an absorbance of 1.1 ± 0.01 units at 734 nm. For DPPH assay, an aliquot of beer (150 μL) was mixed with DPPH solution (2850 μL), and the absorbance was read at 515 nm after 24 h in dark. DPPH solution was prepared by dissolving 24 mg DPPH in 100 mL of methanol, and the solution was then diluted adding methanol to obtain an absorbance of 1.1 ± 0.01 units at 515 nm. For the FRAP assay, the working solution was prepared by mixing 100 mL of 300 mM acetate buffer (pH 3.6) and 10 mL of 10 mM TPTZ solution (2,4,6-tripyridyl-s-triazine) in 40 mM HCl, 10 mL of FeCl_3_⋅6H_2_O solution and 12 mL of distilled water. An aliquot (150 μL) of beer was mixed with the FRAP working solution (2850 μL) and warmed at 37 °C, at dark, for 30 min. The FRAP reaction mixture of samples was read at 593 nm. Results of all tests were expressed in μmol Trolox equivalents (TE) L^−1^ of sample.

### 2.6. Aldehydes and Diacetyl

Aldehydes and diacetyl were determined by gas chromatography/mass spectrometry in duplicate in the final beer according to De Francesco et al. (2015) [20], which is based on solid-phase microextraction with on-fibre derivatization.

### 2.7. Sensory Profile

The sensory profile was performed by a trained panel (composed of 12 people between the ages of 25 and 47 years) through description analysis according to Analytica EBC method 13.10. The members of the sensory panel were asked to describe flavour relevant to aroma (Fruity/estery, Alcoholic/solvent, Fruity/citrus, Herbal/Floral/Spicy, Malty, Oxidised/aged, Sweet) and flavour relevant to taste (the previously considered and Bitter, Astringent, Body, Linger). For each attribute, a score was assigned ranging from 0 to 9, where 1–3 was low intensity, 4–6 was medium intensity and 7–9 was high intensity. The sensory analysis was performed in duplicate.

### 2.8. Statistical Analysis

The statistical analysis was performed using SigmaPlot software (version 12.0; Systat Software, Inc., San Jose, CA, USA). Different matrices originated from technological and analytical replicates were compared by one-way analysis of variance, and the results were further analysed using the Holm–Sidak test and the Tukey test.

## 3. Results and Discussion

Following the addition of the phenolic-rich extracts, the qualitative parameters were analysed as described in Section 2.2.

The analysis were repeated on a quarterly basis for a total of 6 months, thus defining three-time phases indicated as M0 (beer at the end of the refermentation), M3 (3 months after the refermentation) and M6 (6 months after the refermentation).

Among the qualitative analysis carried out, greater attention was paid to those through which it is possible to identify oxidative phenomena and the possible interaction of phenolic extracts with proteins.

In unpasteurized refermented beers, the apparent attenuation is a parameter that allows monitoring the activity of the yeast during storage. In this case, although the beer was stored at room temperature, no statistically significant differences emerged after 6 months of storage (Table 2).

The colour modification is one of most important beer aging indicators, mainly due to oxidation and consequent degradation of polyphenols and the formation of Maillard compounds during storage, especially at warm storage temperature [21,22]. The untreated sample had the greatest colour increase, raising from 5 to 7.4 EBC after 6 months (Table 1). In the added samples of BTGT and Oxnomore Mash, phenolic extracts constituted by derivatives of tannic acid and ellagic acid, respectively, there was a colour intensity reduction in the fresh beer, mainly due to the partial dragging on the bottom of the melanoidins through precipitation of the unstable tanno–protein complex [23]. Samples added with phenolic extracts showed a reduced colour increase compared to the untreated sample confirming the antioxidant effect. Overall, the colour of the beer had a slight increase when aged at temperatures around 20 °C according to Vanderhaegen et al. [21].

It is difficult to find paper that monitor the foam head during beer aging. In the case of the present work, all beers suffered a loss of foam retention measured by NIBEM. This could be due to the release of yeast proteases [24,25] according to He et al. [26], where the authors found a dramatic head retention difference between pasteurized and unpasteurized beers. Interestingly, the foam of the beers treated with Oxnomore super and BTQ was significantly higher than the other samples, demonstrating a protective effect of some tannins to foam collapse [27,28].

During beer storage, phenolic polymers interact with proteins and form insoluble complexes and hazes [29]. Further, free radicals are haze-forming agents [30]. The use of phenolic-rich extracts is useful also for their ability to act as radical scavengers, and thus, as inhibitors of auto-oxidation processes [28]. In the analysis of haze, the untreated sample recorded a substantial increase in turbidity after 6 months. It should be noted that these beers were unfiltered and therefore the initial value was high when compared to a commercial one that is filtered. In fact, the haze was 1.7 EBC for the untreated sample, while it is common to have a haze below 1 EBC for a clear beer. Untreated beer had the most substantial increase in turbidity from 1.7 to 4.7 EBC, while BTGT and Oxnomore mash values went from 1.4 to 2.5 and 2.6 EBC, respectively (Table 2). This phenomenon could be due to the degradation of some proteins to which the phenolic extracts were bound, and which therefore became available again for reaction with other proteins, causing cloudiness. 

For the samples subjected to the treatment with Oxnomore pro, Oxnomore super and BTQ, a progressive decrease in turbidity over time was found.

The beer treated with Oxnomore pro was found to have the most stable turbidity, even decreasing from 2.3 to 1.1 EBC after 6 months, highlighting the dual ability of some phenolic extracts to act as haze preventing and clarifying agents.

Dissolved oxygen was measured to better understand the antioxidant effect of phenolic extracts. The quantity of dissolved oxygen was found to be below 100 ppb in all beers at the end of the refermentation, thanks to the yeast metabolism. The untreated sample (M0) registered the lowest percentage decrease compared to the treated samples, which had a greater reduction of dissolved oxygen depending on their reducing activity. These data confirm that oxidation does not necessarily correspond to consumption of dissolved oxygen. In fact, the ability of phenolic extracts to block oxidized compounds has shifted the balance of the reaction towards the products, thus accelerating the consumption of reagents (oxygen). 

Table 3 reports the analysis of total polyphenols. The addition of phenolic extracts leads to an increase in the total polyphenol content, according to Aerts et al. [28]. In fact, the total polyphenol content is more than double in all beers. As indicated in materials and methods, a dosage of 20 g/hL was used for each extract. After 6 months of storage, the highest amount of polyphenols, however, was found in the beers treated with the condensed tannins Oxnomore pro and Oxnomore super, with 378 mg/L and 461 mg/L, respectively, higher than the 169 mg/L of untreated beer. Another important factor that emerges is the lower intake, despite the same dosage, of polyphenols for BTGT and Oxnomore mash in fresh beer. This unexpected result could be due to a fast reaction of BTGT and Oxnomore extracts with proteins. This reaction did not influence the foam stability, as confirmed by the foam analysis (Table 2). 

The formation of bonds with proteins is confirmed by the clarifying effect in accordance with the haze values (Table 2). Furthermore, the lack of removal of the tanno-protein precipitate allowed that, during the degradation phase of the proteins engaged in this complex, the polyphenols would become available again in solution, generating an increase over the course of storage. 

To better understand the antioxidant effect of phenolic-rich extracts added, three different assays were performed (ABTS, FRAP, DPPH) (Table 4). 

The ABTS, FRAP and DPPH tests indicate that hydrolysable tannins should have a higher antioxidant capacity than condensed tannins in wine model solution [17,30]. To our best knowledge, there are no works on beer. For all three assays, the samples treated with Oxnomore pro and Oxnomore super, two condensed tannins, showed the highest antioxidant power. In particular, the tea tannins Oxnomore are super contrary to that found in wine [17]. The antioxidant activity was higher, if compared with the untreated beer, until the end of the shelf life trials (after 6 months). The antioxidant activity of the extract Oxnomore super was the highest and most stable along the entire trial. BTC and BTQ also showed an interesting and statistically significant antioxidant protection on beer during the 6 months, but that was only confirmed by ABTS and FRAP assays, not by DPPH assay. It could be interesting to underline the protective effect against the oxidation of the condensed extract or mixture of hydrolysable and condensed. The antioxidant assays highlighted the protective potential of some tested tannic extract, in particular the tea extract Oxnomore super. Additional analyses were carried out to better understand the protective effect of phenolic extracts against beer aging.

To this aim, the analyses of aldehydes and diacetyl have been performed. Until a few years ago, the (E)-2-nonenal compound responsible for the cardboard-like off-flavour was considered the main compound responsible for the aging of a lager beer [31]. Today, other key aging aldehydes have been identified, such as furfural, hydroxymethylfurfural, methylfurfural, and lipid oxidation products such as hexanal, heptanal and hexanal, as well as the Strecker aldehyde group: benzaldehyde, methional, 2-methylpropanal, 2-methybutanal, 3-methylbutanal, and phenylacetaldehyde [32,33]. Narziß et al. [34] also discovered a promoting effect of oxygen during beer aging on Strecker aldehydes. Starting from these hypotheses, the addition of tannins should carry out an improvement of beer stability hindering or preventing the formation of staling compounds. Moreover, as stated above, this study concerns refermented beer and so the presence of yeast can also contribute to improve the flavour stability [35]. 

The results of the analyses of aldehydes and diacetyl are shown in Table 5. Acetaldehyde concentrations were higher in fresh beer, contrary to that found by Saison et al. [35], where an increase occurred after 6 months of storage. Surprisingly, the beers treated with phenolic extracts registered a great amount of acetaldehyde after refermentation. This could be due to the tannins that determine an increase in the production of acetaldehyde probably owing to the presence of more structures containing O-diphenols and/or to traces of metal catalysts present as contaminants of tannin preparations [36]. Further, acetaldehyde is formed at the early stage of refermentation by the yeast and is not reduced as long as oxygen is present in the beer [37]. Beer with BTGT, Oxnomore and BTQ had 7214.5, 9849.4 and 6721.1 µg/L of acetaldehyde after refermentation while it had 2945.7, 2523.1 and 3387 µg/L after 6 months of storage. Thus, after 6 months, all beers had similar amounts of acetaldehyde showing a low influence of the phenolic-rich extracts. Strecker aldehydes are compounds widely studied in recent years. 3-methylbutanal and 2-methylbutanal are considered to be responsible for the “malty” character, while methional and phenylacetaldehyde are key compounds of the flavour of “aged” beer [32,38,39]. The concentration of these aldehydes had a positive correlation with the amount of oxygen present in the bottle [32]. In this case, the beers showed an oxygen level below 100 ppb at the end of the refermentation (Table 1) due to the yeast metabolism. Therefore, the development of Strecker aldehydes during storage was very limited, in accordance with other work on beer aging [32]. Herein, the addition of phenolic extracts did not cause a statistically significant reduction in Strecker aldehydes, as shown in Table 5. This result is different from a previous work, where gallotannins appear to inhibit the de novo production of these aldehydes during the whole brewing process, including wort filtration, wort boiling, and clarification [32]. 

Furfural, a key odorant of lager beers aging was found to have a linear increase during the 6 months of storage (Table 5). As with Strecker aldehydes, furfural amount was the same in all beers; after 6 months, an average increase of 80% was recorded, reaching about 30 µg/L. In general, the furfural found was very low compared to work on beer aging [40]. It appears that this aldehyde is very high in aged pasteurized beers or in dark beer, where the value can reach 1000 µg/l or over after 6 months of storage [40,41,42,43], causing sharper, harsher, more lingering bitterness and increased astringency [42]. Another reason could be the warm temperature, as can be seen in work where beer were kept at 40 °C during storage [21]. Here, the lower storage temperature (22–25 °C) could contribute to the reduced formation of furfural. The hexanal, as well as the furfural, increased during aging in a similar manner for all beer. Again, the added tannins seemed to not have an influence on the hexanal content. 

The formation of diacetyl could be due to Maillard reaction and temperature of storage. However, the beers studied were clear and stored between 22 and 25 °C, leading to a low increase in diacetyl throughout the aging. Anyway, diacetyl remained far below the perception threshold for a top fermented beer (100–150 µg/L), with values ranging from 10.1 to 13.3 µg/L after 6 months. The phenolic extracts did not have an influence on the formation of diacetyl.

Sensory analysis is a tool to control beer organoleptic modification during storage. The beers were tasted every 3 months for the aroma and taste, as indicated in Table 6 and Table 7, respectively. The beer was produced with a neutral yeast, Fermentis Safale US-05^®^, that bring out the characteristics of the raw materials rather than those of yeast, allowing the judges to better observe the influence of the addition of phenolic extracts. After 6 months, the aroma profile of beers showed a drop for fruity/estery attribute except for beer with Oxnomore pro. This may be due to the aromatic component of the phenolic tea extract. The citrussy flavour decreased in all beers after 6 months, although it is interesting to note that in fresh beers, Oxnomore mash and BTQ caused a loss of this sensory attribute. Thus, the use of these two extracts might not be appropriate in beers characterized by hops. Also, the herbal/floral/spicy dropped after 6 months. Once again, it should be noted that the phenolic extracts generated aroma changes in the fresh beers. The BTGT enhanced the spicyness, as occurred also for Oxnomore pro, super and BTC. After 6 months, the beers showed an increasing value of the malt notes, according to other works on beer aging, where malt, honey, and caramel aroma notes are often found [2]. The same trend was found by the judges for the taste profile, with a decrease in the fruitiness, citrussy, spicyness, and an increase in malt notes. The aged parameter was not detectable for all beers. 

About the taste profile (Table 7), it can be seen how phenolic extracts influence some organoleptic characteristics. Oxnomore pro increased the citrussy and spicy notes, but above all increased the body of the beer, defined by the judges as more rounded. BTQ increased the malt notes by adding to beer taste crusty bread. Oxnomore mash and BTQ have reduced the bitterness and persistence of fresh beer. Contrary to what was expected, phenolic extracts have only slightly affected the astringency of beer, an aspect linked to the presence of tannins. Only beer treated with Oxnomore super was judged to have higher astringency, mainly due to the condensed tea tannins, which have a strong affinity for binding with proline rich proteins, such as those found in saliva [44]. Overall, the studied beers showed stability like fresh beer after 3 months with a drop after 6 months (Table 7).

## 4. Conclusions

In conclusion, the samples with phenolic extracts showed a better stability in terms of turbidity, colour formation and foam quality.

The use of all studied phenolic extracts showed an enhancement of flavour stability and a protective effect on beer quality. The best results were obtained with the condensed tannins and in particular with the extract Oxnomore super (a high purity green tea extract). 

Specifically, data analysis revealed beer with Oxnomore super as the most stable, limiting the increase in colour and turbidity and the loss of foam. Moreover, the panellists judged the beer added with Oxnomore super to be the most elegant for aroma and taste, with a smooth and rounded body. Further, Oxnomore super showed an interesting antioxidant activity: excellent inhibition of free radicals (DPPH), good reducing capacity against the Fe^3+^ ion (FRAP), and above all exhibited excellent oxygen scavenger action (ABTS) as demonstrated also by the substantial reduction of the dissolved oxygen.

Also, Oxnomore pro recorded relevant antioxidant power and excellent quality and analytical values, but especially improved the overall organoleptic beer profile. Grape seed tannins increased the citric and spicy notes, but above all increased the body of the beer, defined by the judges as more rounded.

This study confirms that the condensed phenolic extracts are a possible solution to counteract the effects of beer aging. According to our results, it is important to test the phenolic extracts before marketing them and then adding beer. This work makes it clear how each tannin can bring different characteristics to beer especially from the organoleptic point of view. Cooperation between universities and companies remains a fundamental aspect.

## Figures and Tables

**Table 1:** Phenolic-rich Extracts added to the Beer.

Commercial Name	Kind of Tannins	Origin	Dosage (g/hL)	Composition on db %
**BTGT**	Hydrolysable	Chinese gall (*Rhus chinensis*)	20	93% of tannic acid
**Oxnomore mash**	Hydrolysable	Quercus gall (*Quercus infectoria*)	20	98.8% of gallic acid
**Oxnomore pro**	Condensed	Grape Seed (*Vitis vinifera*)	20	99.2% of proanthocyanidins
**Oxnomore super**	Condensed	Tea (*Camellia sinensis*)	20	78.4% of Epigallocatechin-3-gallate
**BTC**	Condensed and hydrolysable	*Myrtaceae* spp.	20	99.8% of polyphenols
**BTQ**	Condensed	Quebracho tree (*Schinopsis balansae*)	20	66.0% of polyphenols

**Table 2:** Quality parameters results. Lowercase letters refer to comparison between months of the same sample, while capital letters refer to comparison between the month for each sample. *p* < 0.05. *n* =2. M0 = fresh beer. M3 = three months of storage. M6 = six months of storage.

	Untreated Beer	BTGT	Oxnomore Mash	Oxnomore Pro	Oxnomore Super	BTC	BTQ
ADF (%)							
M0	86.2 aA	84.6 aA	86.0 aA	85.7 aA	85.5 aA	86.0 aA	85.5 aA
M3	85.1 aA	85.3 aA	86.3 aA	85.7 aA	86.1 aA	85.6 aA	85.5 aA
M6	85.5 aA	84.5 aA	85.5 aA	86.2 aA	85.5 aA	87.4 aA	86.0 aA
Colour (EBC-U)							
M0	5.0 aBC	4.4 aA	4.4 aA	5.1 aBC	4.8 aB	5.0 aBC	4.9 aBC
M3	5.4 bB	4.7 bA	4.9 bB	5.8 bC	5.2 aAB	5.2 aAB	5.0 aAB
M6	7.4 cC	6.7 cB	6.2 cA	6.8 cB	6.8 cB	6.8 cB	6.8 bB
NIBEM30 (s)							
M0	290 cD	254 cAB	248 cA	286 cD	278 cCD	280 cCD	267 cBC
M3	237 bB	218 bA	217 bA	258 bCD	270 bD	255 bCD	244 bBC
M6	194 aA	192 aA	195 aAB	215 aC	234 aD	206 aBC	244 aD
Haze (EBC-U)							
M0	1.7 aB	1.4 aA	1.4 aA	2.3 bD	2.1 aC	1.5 aA	2.0 aC
M3	1.4 aA	1.5 aA	1.6 aA	2.0 bB	2.0 aB	1.3 aA	1.9 aB
M6	4.7 bE	2.5 bD	2.6 bD	1.1 aA	1.9 aB	2.2 bC	1.9 aB
Dissolved Oxygen (ppb)							
M0	80.5 bF	72.2 cE	58.0 cD	42.0 cB	58.7 cD	53.0 cC	23.2 bA
M3	31.5 aC	28.0 bB	38.2 bD	21.0 bA	48.2 bE	32.0 bC	20.0 abA
M6	31.7 aD	14.2 aB	11.1 aA	13.3 aB	13.6 aB	18.3 aC	18.3 aC

**Table 3:** Total polyphenols of beers during storage. Capital letters refer to comparison between months of the same sample, while lowercase letters refer to comparison between samples of the same month. *p* < 0.05. *n* = 2. M0 = fresh beer. M3 = 3 months of storage. M6 = 6 months of storage.

Sample/Months	M0 (mg/L)	M3 (mg/L)	M6 (mg/L)
Untreated beer	203.5 aA	166.0 aA	169.0 aA
BTGT	288.0 cA	296.5 bB	300.5 bB
Oxnomore mash	246.0 bA	327.0 dB	309.0 bB
Oxnomore pro	417.0 eA	390.0 eA	378.0 cA
Oxnomore super	489.5 fB	488.0 fB	461.0 dA
BTC	278.5 cA	310.5 bcC	300.5 bB
BTQ	313.5 dA	319.5 cdA	306.0 bA

**Table 4:** Antioxidant activity levels measured by the radical cation analysis (ABTS), ferric-reducing antioxidant power analysis (FRAP) and DPPH. Capital letters refer to comparison between months of the same sample, while lowercase letters refer to comparison between samples of the same month. *p* < 0.05. *n* = 2. M0 = fresh beer. M3 = 3 months of storage. M6 = 6 months of storage.

	Sample/Months	M0 (µM TE/L)	M3 (µM TE/L)	M6 (µM TE/L)
**ABTS**	Untreated beer	3281.9 aA	2385.1 aB	2571.7 bB
	BTGT	3451.0 abA	2880.2 cB	2692.5 bcC
	Oxnomore mash	3265.8 aA	2689.8 bcB	2272.3 aC
	Oxnomore pro	3738.3 cA	3295.0 dB	2936.1 dC
	Oxnomore super	5045.9 dA	4631.6 eB	4860.4 eAB
	BTC	3606.2 bcA	2494.4 abB	2193.2 aC
	BTQ	3479.7 bA	2661.6 bcB	2840.0 cdB
**FRAP**	Untreated beer	1507.9aA	1104.0aB	1202.0aB
	BTGT	2055.9bcdA	1671.4bcBC	1676.8cC
	Oxnomore mash	1752.2abA	1425.0bB	1450.5bB
	Oxnomore pro	2350.8dA	1918.7cB	2025.8dB
	Oxnomore super	3056.4eA	2805.8dAB	2601.1eB
	BTC	2015.4bcA	1507.2bB	1466.1bcB
	BTQ	2144.6cdA	1699.5bcB	1646.0bcB
**DPPH**	Untreated beer	1349.3aA	795.6aB	1127.4aC
	BTGT	1465.2aA	1197.8cA	1248.5aA
	Oxnomore mash	1480.2aA	1001.0bB	1171.6aB
	Oxnomore pro	1711.9bA	1137.0bcB	1290.2aB
	Oxnomore super	2147.3cA	1752.1dB	1821.6bB
	BTC	1341.0aA	1168.0bcA	1154.5aA
	BTQ	1333.0aA	1148.4bcB	1121.1aB

**Table 5:** Aldehydes and diacetyl level during storage. Capital letters refer to comparison between months, while lowercase letters refer to comparison between samples of the same month. *p* < 0.05. *n* = 2. M0 = fresh beer. M3 = 3 months of storage. M6 = 6 months of storage. AA = Acetaldehyde, 2MB = 2-methylbutanal, 3MB = 3-methylbutanal, 2F = 2-furfural, PH = phenylacetaldehyde, HE = hexanal, ME = methional, DI = diacetyl. Nd = not detectable.

	µg/L	AA	2MB	3MB	ME	PH	2F	HE	DI
M0	Untreated beer	4821.8 bD	0.8 aA	4.9 aB	nd	3.1 aA	5.8 aA	0.4 aA	nd
	BTGT	7214.5 dF	0.6 aA	5.6 bB	nd	1.9 aA	5.8 aA	0.3 aA	nd
	Oxnomore mash	3458.1 aBC	nd	2.0 aA	nd	1.5 aA	5.7 aA	0.2 aA	nd
	Oxnomore pro	9489.4 eI	2.8 bBC	14.7 cE	nd	9.9 bC	5.9 aA	0.4 aA	nd
	Oxnomore super	3422.6 aBC	nd	1.6 aA	nd	3.5 aA	5.9 aA	0.3 aA	nd
	BTC	3314.7 aBC	nd	2.7 aA	nd	3.3 aA	5.9 aA	0.2 aA	nd
	BTQ	6721.1 cE	0.4 aA	6.5 bB	nd	5.2 aA	6.2 aA	0.4 aA	nd
M3	Untreated beer	2620.0 bAB	1.8 aB	8.4 abC	3.6 aA	7.8 abB	18.3 aB	0.8 aB	5.4 aA
	BTGT	3386.7 cBC	2.1 aB	8.6 abC	3.2 aA	5.5 aA	18.5 aB	0.7 aB	5.3 aA
	Oxnomore mash	2234.0 aA	1.2 aB	5.7 aB	3.4 aA	5.9 aA	17.2 aB	0.6 aB	5.1 aA
	Oxnomore pro	3569.0 cC	2.7 aBC	12.9 bE	3.0 aA	9.9 bC	15.8 aB	0.7 aB	7.6 aA
	Oxnomore super	2102.0 aA	1.8 aB	6.6 aB	2.9 aA	5.7 aA	16.1 aB	0.7 aB	5.1 aA
	BTC	2122.0 aA	1.6 aB	7.3 aBC	3.4 aA	7.0 aAB	17.5 aB	0.8 aB	6.0 aA
	BTQ	3354.0 cBC	2.3 aB	10.1 bC	3.9 aA	8.1 abB	16.5 aB	1.3 aB	6.7 aA
M6	Untreated beer	2944.0 aB	3.5 aC	11.8 aD	7.3 aB	12.5 aD	30.7 aC	1.1 aB	10.8 bB
	BTGT	2945.7 aB	3.5 aC	11.6 aD	6.4 aB	9.2 aC	33.3 aC	1.1 aB	10.7 bB
	Oxnomore mash	2889.7 aB	2.4 aB	9.9 aC	6.7 aB	10.2 aC	29.4 aC	1.1 aB	10.1 bB
	Oxnomore pro	2523.1 aAB	2.7 aBC	11.1 aD	6.1 aB	9.9 aC	26.0 aC	1.1 aB	15.2 bB
	Oxnomore super	2864.9 aB	3.6 aC	11.5 aD	5.8 aB	7.9 aB	26.3 aC	1.0 aB	10.1 bB
	BTC	3037.8 aBC	3.2 aC	11.8 aD	6.7 aB	10.6 aC	29.6 aC	1.4 aB	12.1 bB
	BTQ	3387.1 bBC	4.1 aC	13.7 aE	7.7 aB	10.9 aC	32.2 aC	2.2 bC	13.3 bB

**Table 6:** Results of aroma profile of beers after six months of storage. n=2. M0= fresh beer. M3= 3 months of storage. M6 = 6 months of storage. Nd = not detected. Score scale 0–9 where 0 = absent, 1–3 = low, 4–6 = medium, 7–9 = high. The values are the mean of 12 tasting reports. FR = fruity/estery, AL = alcoholic/solvent, CI = citrussy. EFS = herbal/floral/spicy, M = malt, A = aged, SW = sweet.

		FR	AL	CI	EFS	M	A	SW
M0	Untreated beer	7	5	6	5	2	0	3
	BTGT	8	3	6	8	2	0	3
	Oxnomore mash	6	3	4	6	2	0	2
	Oxnomore pro	8	2	6	7	0	0	2
	Oxnomore super	7	3	6	7	4	0	2
	BTC	7	2	6	7	3	0	3
	BTQ	4	2	3	4	5	0	2
M3	Untreated beer	7	4	5	5	2	0	3
	BTGT	7	3	5	7	3	0	3
	Oxnomore mash	6	2	4	6	2	0	2
	Oxnomore pro	7	2	6	6	0	0	3
	Oxnomore super	6	2	6	7	4	0	2
	BTC	7	3	5	6	3	0	2
	BTQ	4	2	3	4	4	0	3
M6	Untreated beer	4	3	3	3	4	0	4
	BTGT	4	3	3	5	5	0	4
	Oxnomore mash	4	3	2	4	5	0	4
	Oxnomore pro	6	3	3	5	3	0	3
	Oxnomore super	4	2	3	4	5	0	3
	BTC	5	3	4	5	4	0	5
	BTQ	2	2	2	3	6	0	3

**Table 7:** Results of taste profile of beers after six months of storage. *n* = 2. M0 = fresh beer. M3 = 3 months of storage. M6 = 6 months of storage. Nd = not detected. Score scale 0–9 where 0 = absent, 1–3 = low, 4–6 = medium, 7–9 = high. M6 = 6 months of storage. FR = fruity/estery, AL = alcoholic/solvent, CI = citrussy. EFS = herbal/floral/spicy, M = malt, A = aged, SW = sweet, BI = bitterness, AS = astringency, BO = body, LI = lingering.

		FR	AL	CI	EFS	M	A	SW	BI	AS	BO	LI
M0	Untreated beer	8	3	5	7	2	nd	3	8	3	5	7
	BTGT	7	2	4	7	3	nd	3	7	2	4	7
	Oxnomore mash	6	2	4	6	5	nd	3	6	2	4	6
	Oxnomore pro	8	2	7	8	2	nd	2	8	2	7	8
	Oxnomore super	7	4	6	7	5	nd	3	7	4	6	7
	BTC	7	2	5	8	2	nd	2	7	2	5	8
	BTQ	5	3	4	3	5	nd	2	5	3	4	3
M3	Untreated beer	7	2	4	7	2	nd	3	7	2	4	6
	BTGT	7	2	5	7	3	nd	3	8	3	5	7
	Oxnomore mash	6	2	4	6	5	nd	4	6	2	4	6
	Oxnomore pro	8	3	7	7	2	nd	2	8	3	7	8
	Oxnomore super	7	5	6	7	4	nd	3	7	5	6	7
	BTC	6	2	5	7	2	nd	3	7	2	4	7
	BTQ	5	3	4	3	5	nd	2	5	3	5	3
M6	Untreated beer	5	nd	3	5	5	nd	3	5	nd	3	5
	BTGT	5	nd	2	5	5	nd	4	5	nd	2	5
	Oxnomore mash	4	nd	2	4	5	nd	4	4	nd	2	4
	Oxnomore pro	6	nd	4	6	3	nd	3	6	nd	4	6
	Oxnomore super	4	2	3	4	7	nd	4	4	2	3	4
	BTC	5	nd	3	5	4	nd	3	5	nd	3	5
	BTQ	2	nd	2	3	7	nd	2	2	nd	2	3
